# Debate: can we achieve universal health coverage without a focus on disability?

**DOI:** 10.1186/s12913-018-3547-2

**Published:** 2018-09-26

**Authors:** Hannah Kuper, Johanna Hanefeld

**Affiliations:** 1 International Centre for Evidence in Disability, London School of Hygiene & Tropical Medicine, London, UK; 2 Department of Global Health and Development, London School of Hygiene & Tropical Medicine, London, UK

**Keywords:** Universal health coverage, Health system, Disability

## Abstract

**Background:**

The achievement of Universal Health Coverage (UHC) is a key aim of the global health agenda, and an important target of the Sustainable Development Goals. There is increasing recognition that some groups may fall behind in efforts to achieve UHC, including the 1 billion people globally living with disabilities. A fundamental question for debate is – can UHC be achieved without the inclusion of people with disabilities?

**Main text:**

People with disabilities are more likely to experience poor health. They will therefore have greater need for general healthcare services, as well as rehabilitation and specialist services, related to their underlying impairment. People with disabilities also frequently face additional difficulties in accessing healthcare, incur greater costs when seeking healthcare and often report experiencing worse quality services than others. As a consequence of these different challenges, people with disabilities face specific and added difficulties across three dimensions of UHC: coverage, access to services needed, and at reasonable cost. A focus on people with disabilities is therefore essential to achieving UHC, particularly since they constitute 15% of the global population. To ensure the realisation of UHC is inclusive of and addresses the needs of people with disabilities, health systems need to adapt. A twin-tracked approach is recommended, which means that there is a focus on including people with disabilities in mainstream services, as well as targeting them with specific services needed. There also must be efforts to improve the quality of services (e.g. through healthcare staff training) and enhance cost protection for people with disabilities (e.g. through social protection). A key challenge to changing UHC strategies to be more inclusive is the lack of evidence on what is needed and works, and more research is needed urgently on this topic.

**Conclusions:**

It will be difficult to achieve UHC without a focus on people with disabilities. Changes made to improve coverage for people with disabilities will likely benefit a wider group, including older people, ethnic minorities, and people with short-term functional difficulties. Disability-inclusive strategies will therefore improve health system equity and ensure that we “*Leave no one behind*” as we move towards UHC.

## Background

Universal Health Coverage (UHC) means ensuring access to health services for the whole population, including all the services that they need (promotive, preventive, curative, rehabilitative and palliative), without incurring financial hardship. The achievement of UHC is a key aim of the global health agenda, and is recognised as an important target of the Sustainable Development Goals. Actions to meet UHC include increasing the breadth of the population covered, the depth of services available, and providing increased financial protection. At the heart of UHC is the commitment to equity. It is therefore important to monitor equity impact as countries move towards implementing UHC, and this is included within the Monitoring and Evaluation Framework for UHC [1]. While there is increasing recognition that some groups, like older people or ethnic minorities, may fall behind in efforts to achieve UHC [2], there has been limited attention to date on how to ensure policies to move towards UHC are inclusive and responsive for people living with disabilities. This is an important gap, since there are approximately 1 billion people with disabilities, making up 15% of the world’s population [3]. In coming years, disability is likely to become even more common as the global population ages, health behaviours worsen, and disabling chronic diseases like stroke and dementia prevail [4]. A fundamental question for debate is – can UHC be achieved without the inclusion of people with disabilities?

## Main text

There is good evidence that people with disabilities experience poorer health on average than those without disabilities, and this can occur through several different pathways [3, 5]. People with disabilities include those who have long-term physical, mental, intellectual or sensory impairments which in interaction with various barriers may hinder their full and effective participation in society on an equal basis with others [6]. By definition therefore, people with disabilities have underlying impairments (e.g. visual impairment) and health conditions (e.g. epilepsy) which can create needs for healthcare. People with disabilities may also have poorer general health for a variety of other reasons, such as difficulties in accessing healthcare, vulnerable living conditions, and adverse health behaviours [7]. They are also on average older, poorer, less likely to work and have lower life satisfaction [8, 9], all linked to poor physical and mental health. For all these reasons, people with disabilities will often have higher healthcare needs overall [3, 5, 10], and will require good general healthcare, covering the full spectrum from preventive services and health promotion, through primary, secondary and tertiary care. They may also need rehabilitation and specialist treatment - related to their underlying impairment – in order to “optimize functioning and reduce disability” [11]. These services include medication, surgery, assistive devices (e.g. hearing aids), and therapeutic rehabilitation (e.g. physical therapy).

Across the world, there is extensive qualitative evidence that people with disabilities face difficulties in accessing healthcare [3, 5]. Potential barriers include discrimination, physical inaccessibility, and information inaccessibility. Another key concern is that people with disabilities often incur greater costs when seeking healthcare, yet are on average poorer, and so are more likely to incur catastrophic health expenses [3], which may drive them further into poverty. Taking the example of children with Congenital Zika Syndrome, which has been a focus of our recent research, these children experience multiple health conditions including visual impairment, epilepsy, feeding difficulties and joint problems, and so need help from a broad range of doctors and specialists. At the same time, they still need vaccinations and other routine care (e.g. for normal childhood illnesses), but general practice physicians may not feel confident to treat them. As a result, these children require repeated visits to specialist services, often located far from their homes. These extensive healthcare needs are particularly challenging as the children are on average from poorer families, which makes access even more challenging and places a financial burden on families.

It is also clear that people with disabilities often report experiencing worse quality services than others, for instance due to a lack of skill and knowledge of health professionals or absence of accessible equipment [3, 5]. This is important, given the growing evidence on the role of quality in ensuring increased utilisation of health services. As a consequence of these different challenges, people with disabilities face specific and added difficulties across three dimensions of UHC: coverage, access to servicess needed, and at reasonable cost. Integrating a gender lens is important, as with our understanding of health systems overall [12]. Women with disabilities may be particularly disadvantaged, as they may have additional healthcare needs (e.g. related to reproductive health), lower access to finances, and less independence in seeking care.

A focus on people with disabilities is therefore essential to achieving UHC, as they are a large group, often not covered fully by health services, and vulnerable to incurring financial hardship. The challenges to accessing equitable healthcare experienced by people with disabilities may also be encountered by other poor and vulnerable groups, and so achievement of health coverage for people with disabilities can be seen as a marker of equity in the whole health system [13]. Appropriate access is also a human rights issue. The right to healthcare among people with disabilities is well established in international law and the United Nations Convention on the Rights of Persons with Disabilities [6], as well as in the laws of most countries. Yet, the limited literature that is available shows that there is a large gap between policy/laws and practice [3]. Providing good access to healthcare services for people with disabilities will ensure that their rights are met and help in achieving good health, with consequent benefits in terms of improved well-being, life satisfaction and reduced morbidity, mortality and poverty. Yet, a recent large study from Afghanistan showed that investments towards UHC led to very limited or no improvement in services for people with disabilities [14]. Research from Chile and Greece suggests that reforms in the health system to promote UHC have shifted the focus from human rights indicators to economic indicators, which have left people with disabilities further behind in access to healthcare [15]. This evidence suggests that without specific attention on people with disabilities, the opportunity provided by UHC will be lost.

To ensure the realisation of UHC is inclusive of and addresses the needs of people with disabilities, health systems need to adapt. But, how should this be done? A twin-tracked approach is recommended to address the needs of people with disabilities. This approach means that there is a focus on including people with disabilities in mainstream services, as well as targeting them with specific services needed. Including people with disabilities in mainstream health services requires a focus on improving access to health care (e.g. providing accessible facilities, outreach services or assistance in transport) and ensuring that the full range of services needed are provided (e.g. ensuring that women with disabilities receive sexual health services). Specific services required by people with disabilities include rehabilitation and assistive devices. Here, there are often major gaps, as the services are frequently not widely available or provided only through NGOs. Efforts are being made to scale up access to rehabilitation and assistive devices, such as through WHO’s REHAB 2030 programme, or WHO’s Global Cooperation on Assistive Technology initiative, which is working to improve access to high-quality affordable assistive products globally. Scaling up these specialist services can help to improve functioning among people with disabilities, but also be beneficial to people with non-communicable diseases or other temporary conditions (e.g. fractures and injuries) to avoid becoming disabled. As well as scaling up services, we may also need to think of innovative methods for providing rehabilitation and specialist services, like running parent support programmes to help parents address the needs of their child with Congenital Zika Syndrome.

There also needs to be a focus on improving quality of services. Mechanisms for this include the further training of health care providers on the needs of people with disabilities as well as ensuring that accessible equipment is available. Policies and laws should be in place that ensure that healthcare services are both inclusive and good-quality, and that disability-awareness is incorporated into training of healthcare professionals. Cost protection for people with disabilities is another important concern, and this needs to be incorporated or explicitly considered when designing strategies for financial protection in UHC. One mechanism to reduce financial vulnerability is to include financial protection for healthcare within the widely available social protection programmes for people with disabilities. For instance, in Vietnam, some people who receive the Disability Allowance also receive free health insurance, to help mitigate their high household healthcare expenditure. This also happens in Brazil, where the families of children with Congenital Zika Syndrome receive financial benefits to help offset some of the costs incurred in caring for their child.

A key challenge to changing UHC strategies to be more inclusive is the lack of evidence on what is needed and works. Tools currently available to monitor UHC are relatively simplistic and focus more on access than coverage [16], and so are not appropriate to identify the added needs of people with disabilities. More research is required to identify the gaps and solutions in achieving UHC for people with disabilities. Another issue is that different barriers and challenges will exist in different settings, and so solutions need to be locally developed. A key slogan of the Disability Movement is *“Nothing about us, without us”*, and so people with disabilities must be included in the design of these strategies to ensure that they are feasible and acceptable. Local Disabled People’s Organizations (DPOs) can play an important role in this respect, raising the voices of people with disabilities and helping to build an inclusive healthcare system.

## Conclusions

It will be difficult to achieve UHC without a focus on people with disabilities. On the positive side, changes made to improve coverage for people with disabilities, such as better physical accessibility and improved skills of health professionals to communicate with a range of clients, will likely benefit a wider group, including older people, ethnic minorities, children, and people with short-term functional difficulties. Disability-inclusive strategies will therefore help the health system to be more responsive to diversity, and so cover a variety of population groups. These changes will improve equity in the health system overall and make sure that we “*Leave no one behind*” as we move towards UHC.

## Abbreviation

UHC: Universal Health Coverage
